# The soybean plasma membrane GmDR1 protein conferring broad-spectrum disease and pest resistance regulates several receptor kinases and NLR proteins

**DOI:** 10.1038/s41598-024-62332-4

**Published:** 2024-05-28

**Authors:** Micheline N. Ngaki, Subodh K. Srivastava, Wang Feifei, Madan K. Bhattacharyya

**Affiliations:** 1 Department of Agronomy, Iowa State University, Ames, IA 50011 USA; 2 Department of Entomology and Plant Pathology, North Carolina State University, Raleigh, NC 27695 USA; 3 Present Address: USDA-ARS APDL, BARC-East Building 1040, 10300 Baltimore Ave., Beltsville, MD 20705 USA; 4 Northeast Institute of Geography and Agroecology, Key Laboratory of Soybean Molecular Design Breeding, The Chinese Academy of Sciences, Harbin, 150081 China

**Keywords:** Biotic stress, Broad-spectrum disease resistance, Soybean, *GmDR1* receptor, Chitin, RNA-seq, Plant Basal resistance, NB-LRR proteins, Transcriptomics, RT-qPCR, Biotechnology, Genetics, Molecular biology, Plant sciences

## Abstract

Overexpression of *Glycine max disease resistant 1* (*GmDR1*) exhibits broad-spectrum resistance against *Fusarium virguliforme*, *Heterodera glycines* (soybean cyst nematode), *Tetranychus urticae* (Koch) (spider mites), and *Aphis glycines* Matsumura (soybean aphids) in soybean. To understand the mechanisms of broad-spectrum immunity mediated by *GmDR1*, the transcriptomes of a strong and a weak *GmDR1*-overexpressor following treatment with chitin, a pathogen- and pest-associated molecular pattern (PAMP) common to these organisms, were investigated. The strong and weak *GmDR1*-overexpressors exhibited altered expression of 6098 and 992 genes, respectively, as compared to the nontransgenic control following chitin treatment. However, only 192 chitin- and 115 buffer-responsive genes exhibited over two-fold changes in expression levels in both strong and weak *GmDR1-*overexpressors as compared to the control. MapMan analysis of the 192 chitin-responsive genes revealed 64 biotic stress-related genes, of which 53 were induced and 11 repressed as compared to the control. The 53 chitin-induced genes include nine genes that encode receptor kinases, 13 encode nucleotide-binding leucine-rich repeat (NLR) receptor proteins, seven encode WRKY transcription factors, four ethylene response factors, and three MYB-like transcription factors. Investigation of a subset of these genes revealed three receptor protein kinases, seven NLR proteins, and one WRKY transcription factor genes that are induced following *F. virguliforme* and *H. glycines* infection. The integral plasma membrane GmDR1 protein most likely recognizes PAMPs including chitin and activates transcription of genes encoding receptor kinases, NLR proteins and defense-related genes. GmDR1 could be a pattern recognition receptor that regulates the expression of several NLRs for expression of PAMP-triggered immunity and/or priming the effector triggered immunity.

## Introduction

In the early step of plant defenses, molecular patterns associated with the invading pathogen and pests are recognized by the plant plasma membrane-localized pattern-recognition receptors (PRRs) including receptor-like kinases (RLKs) or receptor-like proteins (RLPs)^1^. It has been documented that PRRs recognize microbe- (or pathogen-) associated molecular patterns (MAMPs or PAMPs) including the bacterial flagellin, elongation factor-Tu and peptidoglycan, and the fungal polysaccharide chitin and the oomycete glucan to activate the first layer of plant immunity known as pattern-triggered immunity (PTI)^2,3^.

Among the plant PAMPs, the fungal cell wall feature and linear polymer of N-acetyl-D-glucosamine chitin is an important activator of the plant immunity and defense mechanisms^4–6^. PAMPs such as chitin and glucan are liberated by the plant chitinases and glucanases through degradation of fungal walls during pathogen invasion^6,7^. Multiple reactions are induced by these cell wall components including the expression of defense-related genes following recognition of these PAMPs by the PAMP recognition receptors^7,8^.

The second layer of defense, the effector-triggered immunity (ETI), is regulated by intracellular nucleotide-binding domain and leucine-rich repeat (NLR) containing receptors termed resistance (R) proteins that recognize the pathogen effector proteins^1,9,10^. Plant genome contains hundreds of genes encoding NLR proteins that play an important role in the perception of pathogen effectors and conferring race- or gene-specific disease resistance^11–13^. The nucleotide-binding (NB) domains bind nucleotides and contain highly conserved motifs including kinase-2, P-loop, GLPL or Gly-Leu-Pro-Leu, MHD motifs^14–16^. The leucine-rich repeat (LRR) domain is involved in effector recognition that determine the expression of race- or gene-specific resistance^17^. The N-terminal region of NLR proteins possess a Toll/interleukin-1 receptor (TIR) domain in TIR-NLR (TNLR) proteins or a coiled-coil (CC) domain in CC-NLR (CNLR) proteins^18,19^.

Despite the fact that innumerable studies have dissected PTI, ETI, and their downstream signaling pathways regulated by the plant hormones, salicylic acid (SA), jasmonate (JA), and ethylene (ET), much is unknown about the mechanisms of broad-spectrum resistance against multiple pathogens and pests regulated by PAMP recognition receptors. In the United States, over a dozen pathogens routinely cause annual soybean yield suppression valued close to $4 billion^20^. The soybean cyst nematode (*H. glycines*) annually suppresses soybean yield valued at over $1.1 billion and *Fusarium virguliforme* over $0.4 billion^20^. *F. virguliforme* causes sudden death syndrome (SDS) and rapidly suppresses accumulation of transcripts of a limited number of genes including *Glycine max disease resistance 1* (*GmDR1*)^21^. Its overexpression enhances resistance of soybean against the *H. glycines,* and two insect pests, spider mites (*Tetranychus urticae*, Koch) and soybean aphids (*Aphis glycines* Matsumura) in addition to *F. virguliforme*^22^. This study suggested that *GmDR1*-mediated broad-spectrum resistance could be initiated through perception of the PAMP chitin found in all four-soybean pathogen and pests. Chitin has been demonstrated to interact with receptor complex CERK1 and LYK5 RLKs^23,24^. In rice, the RLP chitin-elicitor binding protein CEBiP recognizes chitin^5^. In soybean, the transmembrane cysteine-rich receptor-like kinases (CRKs) involved in plant immunity binds to chitin^25^. In this study, a transcriptomic approach was applied to investigate the molecular basis of broad-spectrum resistance mediated by overexpression of *GmDR1*. Among the identified 64 biotic stress-related related genes 53 were induced and 11 repressed by chitin. The chitin-induced genes include those that encode nine receptor kinases, 13 NLR-type receptors including 10 TNLR and three CNLR, seven WRKY transcription factors, four ethylene response factors, and three myeloblastosis (MYB)-like proteins. Investigation of a subset of these chitin-induced genes uncovered three receptor protein kinases, seven NLR proteins, and one WRKY transcription factor genes that are also induced by the *F. virguliforme* and *H. glycines* infection. These observations suggest that *GmDR1* is a master-regulator of several NLR-type disease resistance genes that facilitate the expression of broad-spectrum disease resistance governed by the overexpressed *GmDR1* gene in soybean.

## Materials and methods

### Soybean plant material, growing conditions, and inoculation

Three soybean lines were used in this study are the non-transgenic soybean cultivar Williams 82 (W82), and two *GmDR1* transgenic lines (*GmDR1*-overexpressors). The transgenic lines DR1-136, the weak *GmDR1*-overexpressor (Promoter 2-*GmDR1*), and DR-107, the strong *GmDR1*-overexpressor (Promoter 3-*GmDR1*), were previously generated in the cultivar Williams 82 background^22^. The soybean plants were grown on Berger BM7 soil that is composed of Sphagnum peat moss (coarse) (63–69%), pine bark (23–27%), perlite (8–12%), wetting agent (ethoxylated alkylphenol), dolomitic and calcitic limestone in a growth chamber maintained at the constant temperature of 23^◦^C and a photoperiod of 16 h of light/ 8 h of dark.

For the root-inoculation experiment, the seeds were sown on soil/sand mixture carrying *F. virguliforme* inocula as previously described^21,26^. The soybean seeds of *GmDR1* overexpressors DR1-136 and DR1-107, and nontransgenic Williams 82 (W82) were sown in a 1:1 mixture of sand:soil inoculated with *F. virguliforme* inoculum at a ratio of 1: 20:: inoculum: soil mix as reported earlier^21,26^.

For *H. glycines* inoculation, seeds were planted to soil collected from Muscatine, Muscatine County, Iowa, containing ~ 50 cysts of the *H. glycines* HG type 2.5.7 (Race 5)^27^. For *H. glycines* infection, seeds were sown in cone-tainers filled with the Muscatine soil and grown in a water bath set at 27 °C under natural light conditions for two weeks^22,27^. Apart from the cyst formation, the soybean plants remained healthy and did not show any obvious disease symptoms.

### The source of soybean lines

The cultivar Williams 82 was obtained from the USDA Soybean Germplasm Collection. The contact person for the seeds is Adam Mahan (adam.mahan@usda.gov), Geneticist, USDA/ARS Soybean Germplasm Collection, 1101W. Peabody Drive., National Soybean Research Center, Urbana, IL 61801, USA. The transgenic lines were generated and characterized in the previous studies^22^.

The materials studied in this research are available from the Bhattacharyya lab, G319 Agronomy Hall, Iowa State University, Ames, IA 50011, USA. All plant collection methods complied with relevant institutional, national, and international guidelines and legislation of the International Union for Conservation of Nature.

### Chitin treatment

At the first trifoliate stage, two weeks after planting, the seedlings were used for an experiment with technical grade chitin azure, a chitinase substrate (Sigma-Aldrich, cat# C3020). A modified stem-cut method was used to administer the chitin treatment^28^. For each genotype, nine seedlings were treated with chitin and nine were with buffer in each experiment. We conducted three experiments. In half of the chitin treatment, the stem-cuts received 0.5 mL of 100 µmol/L chitin in sodium phosphate (15 mmol/L, pH 6.5); and in controls, stem-cuts received 0.5 mL of sodium phosphate buffer (15 mmol/L, pH 6.5) only. The stem-cuts were placed in 2-ml Eppendorf tubes containing either chitin suspension or phosphate buffer. After the treatment suspensions were depleted, all stem-cuts were transferred to 50-ml tube containing 25 ml distilled water and incubated in a growth chamber maintained at 22.5 °C, 16 h light (85 µmol m^−2^ s^−1^) and 8 h dark photoperiod. Leaf samples were harvested at 12 and 24 h post-treatment. Collected leaves were immediately placed in liquid nitrogen and stored at − 80 °C for RNA isolation.

### RNA extraction and reverse transcriptase quantitative polymerase chain reaction (RT-qPCR) analysis

The transgenic and nontransgenic Williams 82 plants were infected with *F. virguliforme* and infected root samples were harvested 14 days following inoculation and used for RNA isolation. Root samples of *H. glycines* infected plants were harvested 15 days after planting and used for RNA extraction. Leaves of the soybean stem cuts 12 and 24 h following chitin treatment were used for RNA preparation. The total RNA samples were extracted from leaves or roots of nontransgenic Williams 82, DR1-136, and DR1-107 lines using the SV total RNA Isolation System that includes on-column DNase treatment (Promega, Inc., Madison, WI, USA). The quality of RNAs was assessed on agarose gel and NanoDrop microvolume spectrophotometer (Thermo Scientific, Waltham, MA, USA).

A reverse transcription kit (Superscript III First-strand synthesis SuperMix, Invitrogen by life technology) was used to synthesize first-strand cDNA from 1–2 μg total RNAs of each RNA sample. The sequences of selected 28 differentially expressed genes (DEGs) were retrieved from Phytozome v13 and SoyBase for designing primers^29,30^. The primers used in the RT-qPCR are listed in Table S1. The soybean *Elongation factor 1-b* (*ELF1-b*) gene (*Glyma.02g44460*) was used as an internal control in RT-qPCR. The reaction was carried out in a 96-well microtiter plate on an iQ5 Biorad instrument using SYBR Green Master Mix, Applied Biosystems, by life technologies (Austin, TX). The relative expression levels of genes were evaluated using the 2^−ΔΔCT^ method^31^. Three biological repeats, each comprising three technical replications, were conducted.

### RNA-sequencing, read mapping and transcript assembly, identification and differential expression analysis

RNA samples obtained from growth chamber-grown plants were sequenced at the Iowa State University DNA facility, using the HiSeq3000 and Miseq Illumina Next Generation Sequencing platforms (^32^; http://www.heatmapper.ca/). The analysis of RNA Seq was performed using the “Tuxedo suite” that offers a set of tools for analyzing RNA-Seq data. These RNA-seq analysis tools generally fall into three categories: (i) those for read alignment; (ii) those for transcript assembly or genome annotation; and (iii) those for transcript and gene quantification (^32^; http://www.heatmapper.ca/). Statistically significant genes with a *p* greater than the FDR after Benjamini–Hochberg correction for multiple-testing were further analyzed (option “yes”). The generated RNA-seq data set included the FPKM values for each gene. The initial analysis consisted of the comparison between Williams 82 and each of the two transgenic lines, DR1-136, and DR1-107, 12 and 24 h following buffer and chitin treatments. To investigate the chitin induced genes and genes involved in resistance, DEGs between Williams 82 and transgenic lines were identified in each of the specific comparison detailed in Dataset S1.

Next, Perl software (https://www.perl.org/get.html) and Microsoft excel was used to identify commonly up-regulated or down-regulated in both transgenic lines compared to Williams 82 at 12 and 24 h following buffer and chitin treatments (Dataset S1). The lists of DEGs commonly upregulated and downregulated in both transgenic lines were further analyzed in the bioinformatics and evolutionary genetics program (http://bioinformatics.psb.ugent.be/webtools/Venn/), which detected the overlapping DEGs. The DEGs that had an average absolute fold change transcripts levels ≥ 2 in the two transgenic *GmDR1* overexpressers as compared to the nontransgenic Williams 82 were retained for subsequent analysis. The final number of DEGs was therefore reduced to 115 from 143 in the buffer treatment, and to 192 from 289 in the chitin treatment (Datasets S2 and S3).

### MapMan and gene ontology (GO) annotation analyses

To determine gene function, the plant-specific visualization tool, MapMan, was used to identify the DEGs that are involved in specific pathways. The MapMan software was obtained from https://mapman.gabipd.org/ to visualize the DEGs by using the transcriptomic data. The mapping file (Map files/Gmax_189.txt) was downloaded from the MapMan Store (http://mapman.gabipd.org/web/guest/mapmanstore). Then, the annotation gene file (downloaded from https://phytozome.jgi.doe.gov/pz/portal.html; file name: Glycine max Wm82.a2.v1/Gmax_275_Wm82.a2.v1.synonym.txt) was used to transfer gene ID of Gmax_189.txt into gene ID of Gmax_275 version for analyzing the transcriptomic data.

The automated annotations using the soybean sequences allowed the assignment of more than 54,505 gene-classifications into 35 BINs. Amino acid sequences of proteins encoded by the DEGs were obtained from Phytozome^29^ and SoyBase^30^. To identify the conserved domains and annotation descriptions, the amino acid sequences were blasted in NCBI. Gene ontology (GO) annotation analyses of DEGs were conducted based on their molecular functions, biological processes and cellular components using SoyBase GO term enrichment tool.

## Results

### Investigation of the transcriptomes regulated by *GmDR1*

The molecular basis of broad-spectrum pathogen resistance mediated by *GmDR1* against two soybean pathogens, *F. virguliforme* and *H. glycines*, and two pests, spider mites and soybean aphids, was investigated by comparing the transcriptomes of two *GmDR1* overexpressors: (i) weak overexpressor, the DR1-136 line and (ii) strong overexpressor, the DR1-107 line, with that of the (iii) nontransgenic control Williams 82 line following treatment with the PAMP chitin^22^. Among the chitin-induced genes, previously reported chitin-responsive genes^33^ were detected confirming that the chitin treatment was effective (Table S2).

**Figure 1:**
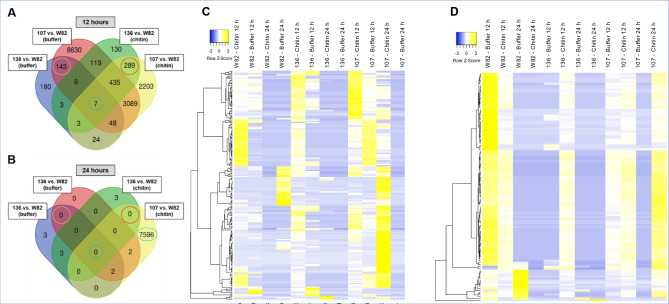
Frequency and expression patterns of differentially expressed genes (DEGs). (**A**) Frequencies of genes that are differentially expressed in the weak (136, DR1-136) and strong (107, DR1-107) *GmDR1*-overexpressers as compared to the nontransgenic Williams 82 (W82) line 12 h following chitin or phosphate buffer (buffer) treatment. The 143 and 289 DEGs, common to both *GmDR1*-overexpressers 12 h following buffer and chitin treatments and with at least 1.5-fold change and *p *$\le$* 0.05* between W82 and each of the *GmDR1*-overexpressers, are shown with orange and red circles, respectively. Expression of seven genes (shown with green circle; Table S3) was significantly altered in *GmDR1*-overexpressers 12 h following chitin and buffer treatments. (**B**) Frequencies of genes that are differentially expressed in the weak (136, DR1-136) and strong (107, DR1-107) *GmDR1*-overexpressors as compared to the nontransgenic W82 line 24 h following chitin or phosphate buffer treatment. (**C**) Heat map displaying the differential expression patterns of the 192 (129 induced and 69 repressed; Dataset S2) chitin-responsive DEGs that were differentially expressed at least by twofold with *p *0.001 in each of the *GmDR1*-overexpressors as compared to W82 12 following chitin treatment. Differential expression levels of these genes 24 h following chitin treatment, and 12 and 24 h following buffer treatment are also presented. (**D**) Heat map displaying the differential expression patterns of the buffer-responsive 115 DEGs (two induced and 113 repressed; Dataset S3) that were differentially expressed at least by 2- fold and *p *0.001 in each of the *GmDR1*-overexpressors as compared to W82 12 following buffer treatment. Differential expression levels of these genes 24 h following buffer treatment, and 12 and 24 h following chitin treatment are also presented.

The gene expression patterns following chitin and buffer treatments were complex. Compared to the nontransgenic Williams 82, more genes were transcriptionally regulated in the strong *GmDR1*-overexpresser DR1-107 compared to the weak *GmDR1*-overexpresser DR1-136 following chitin treatment (Fig. S1B; Dataset S1). Both transgenic lines confer resistance against the fungal pathogen *F. virguliforme*^22^. The number of DEGs in DR-107 was much higher than in DR1-136 when the gene expression levels of the two lines were compared with that of the nontransgenic Williams 82 line 12 h following chitin or buffer treatment (Fig. 1A; Dataset S1). By 24 h, the number of DEGs between DR1-136 and nontransgenic Williams 82 was only three as opposed to 7,596 between DR1-107 and Williams 82 (Fig. 1B).

### Identification of several *GmDR1*-regulated genes associated with the expression of broad-spectrum pathogen and pest resistance

A subset of 289 genes were differentially expressed in both *GmDR1*-overexpressors as compared to Williams 82 12 h following chitin treatment (Fig. 1A). Of these 289 genes, only 192 had ≥ twofold changes in transcript levels (*p* value ≤ 0.001) in both overexpressors compared to Williams 82 (Dataset  S2).

Phosphate buffer treatment led to differential expression of 143 genes at 12 h post treatment (Fig. 1A). However, only 115 genes of the 143 genes had ≥ twofold changes in transcript levels (*p* value ≤ 0.001) in both overexpressors compared to Williams 82 following phosphate buffer treatment (Dataset  S3).

By 24 h post treatments, no DEGs were observed that were common between comparisons of Williams 82 with either DR1-136 or DR1-107 (Fig. 1B). Uncommon DEGs, that were not common to both *GmDR1* overexpressers when transcript levels were compared with that of Williams 82, include only three in the weak *GmDR1*-expresser; whereas over 7000 DEGs in the strong *GmDR1*-overexpresser 24 h following chitin treatment (Fig. 1B). It was observed that only seven genes are differentially regulated between the nontransgenic control and either of the overexpressors, 12 h but not 24 h following chitin and phosphate buffer treatments (Fig. 1A; Table S3).

Heatmap of the selected 192 DEGs between the *GmDR1* overexpressors and nontrangenic control 12 h following chitin treatment revealed 129 DEGs (67%) that were induced, whereas 63 DEGs (33%) that were repressed in both transgenic lines as compared to the nontransgenic control (Fig. 1C, Dataset S2). Heatmap of the selected 115 buffer-responsive DEGs showed that 113 were repressed and only two were induced among the *GmDR1*-overexpressors as compared to Williams 82 following buffer treatment (Fig. 1D, Dataset S3).

### Identification of key genes transcriptionally regulated by overexpressed *GmDR1*

**Figure 2:**
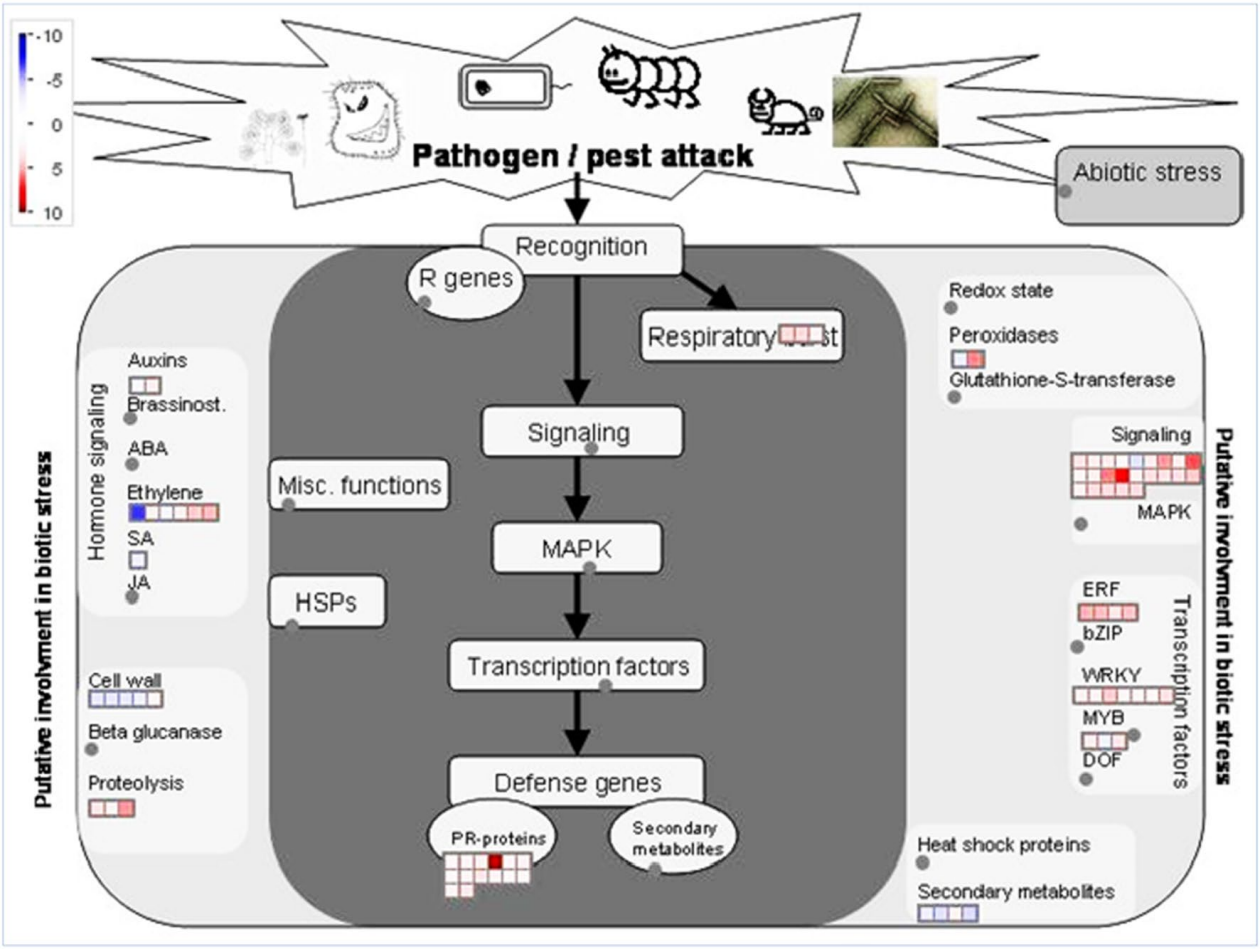
Genes regulated by overexpressed *GmDR1* following treatment with chitin. The 192-chitin responsive DEGs common to both strong and weak *GmDR1* overexpressors (Fig. 1) were visualized using the MapMan software. Among the 192 chitin-responsive genes, 64 are involved in the biotic-stress pathways (Dataset S4A) and 18 in metabolic pathways (Dataset S4B). Blue indicates a decrease, whereas red indicates an increase (log2 fold changes) in the transcription levels of a gene when compared with that of the nontransgenic Williams 82 control.

To identify the defense-related genes and their relative transcript abundance regulated by *GmDR1*, the MapMan program was applied on the selected subsets of 192 and 115 genes showing > twofold changes in transcript levels in both *GmDR1*-overexpressors as compared to that in the nontransgenic Williams 82 control line 12 h following chitin and buffer treatment, respectively. Investigation of the 192 chitin-responsive DEGs revealed 64 biotic stress-related and 18 metabolism pathways-related genes (Fig. 2; Dataset 4A,B). Of the 64 biotic stress-related genes, 53 were upregulated and 11 were downregulated following chitin treatment. Genes encoding 10 receptor kinases, 13 NLR proteins (11 TNLR and two CNLR), seven WRKY transcription factors^34,35^, four ethylene response factors (ERFs)^36^, and three MYB-like proteins^37^ were identified from the 53 upregulated chitin-responsive DEGs (Table 1; Dataset 4A). Among the 115 buffer-responsive DEGs, two abiotic stress-related, 26 biotic stress-related, and seven metabolism pathway-related genes were identified (Fig. S2; Dataset 4C,D).

**Table 1:** Expression levels of the 22 DEGs encoding nine LRR receptor-like kinases and 13 NLR-type disease resistance proteins 12 h following chitin or buffer treatment.

Gene name	Annotation	C3	C4	C5	C6	C7
LRR-receptor kinases						
Glyma.05G126400	LRR Transmembrane Protein Kinase	3.1	3.3	− 2.1	1.1	− 1.1
Glyma.14G118800	LRR receptor-like Ser/Thr- Protein Kinase	4.3	11.8	− 9.2	− 1.3	− 2.0
Glyma.16G182700	LRR Receptor-Like Protein Kinase	5.5	5.3	1.0	1.0	1.2
Glyma.16G182866	LRR receptor-like Ser/Thr-Protein kinase GSO1	4.7	4.1	1.0	1.0	1.4
Glyma.16G184200	LRR Receptor-Like Protein Kinase	2.1	2.7	− 1.3	1.3	1.7
Glyma.16G186100	LRR Receptor-Like Protein Kinase	3.0	9.9	1.2	− 1.1	− 1.2
Glyma.16G186600	LRR Receptor-Like Protein Kinase	3.3	2.7	− 1.1	1.2	1.3
Glyma.16G193600	LRR Receptor-Like Protein Kinase	2.3	3.5	− 1.9	1.6	1.8
Glyma.18G254000	LRR Receptor-Like Protein Kinase	3.3	2.9	− 1.4	− 1.2	− 1.0
NBS-LRR-type disease resistance receptors						
Glyma.07G077700	NB-ARC-LRR, RPS2-like	2.2	2.2	− 1.3	− 1.1	− 1.2
Glyma.17G180300	CC-NBS-LRR, RPW domain	2.4	2.8	1.8	1.8	1.3
Glyma.16G137000	TIR-NBS-LRR class	2.9	4.0	1.0	1.0	− 1.1
Glyma.16G137600	TIR-NBS-LRR class	2.6	3.3	− 2.1	2.0	1.9
Glyma.13G028100	TIR-NBS-LRR class	2.4	3.1	1.1	1.5	1.5
Glyma.16G136600	TIR-NBS-LRR class	2.3	4.1	1.5	1.4	1.2
Glyma.11G153000	TIR-NBS-LRR class	2.1	2.7	− 5.9	− 1.0	1.1
Glyma.12G135600	TIR-NBS-LRR class	2.0	2.1	− 2.2	1.4	1.5
Glyma.03G048500	TIR-NBS-LRR, RPS4-like	5.0	3.1	− 1.0	2.1	2.0
Glyma.03G048200	TIR-NBS-LRR	5.6	4.8	1.0	1.2	1.2
Glyma.09G075500	TIR-NBS-LRR, RPS4-like	2.1	2.1	− 2.9	1.2	1.0
Glyma.03G053500	TIR-NBS-LRR	30.1	9.5	1.0	1.1	1.1
Glyma.03G053602	TIR-NBS-LRR	18.8	8.0	1.0	1.3	1.2
Signaling proteins						
Glyma.17G011700	F-box family protein with a domain of unknown function	2.6	2.3	2.2	2.2	1.2
Glyma.11G003500	Pyridoxal-5\′-phosphate-dependent enzyme family protein	5.3	43.3	− 2.1	− 1.3	− 2.8
Glyma.10G148700	Calmodulin-binding protein	2.9	5.8	− 2.4	− 2.4	1.4
Glyma.04G223200	WRKY DNA-binding protein 55	2.6	6.1	− 4.5	− 1.3	− 1.6
Glyma.04G057700	Integrase-type DNA-binding superfamily protein	3.1	6.2	1.6	1.0	− 1.3
Glyma.03G066800	NAD(P)-linked oxidoreductase superfamily protein	2.2	2.3	2.6	2.0	2.1

C3, Column 3: Fold changes (FCs) in DR1-136 versus W82 12 h following chitin treatment.

C4, Column 4: FCs in DR1-107 versus W82 12 following chitin treatment.

C5, Column 5: FCs in DR1-136 versus W82 12 h following buffer treatment.

C6, Column 6: FCs in DR1-107 versus W82 12 h following buffer treatment.

C7, Column 7: FCs in W82 12 h following chitin versus buffer treatment.

**Figure 3:**
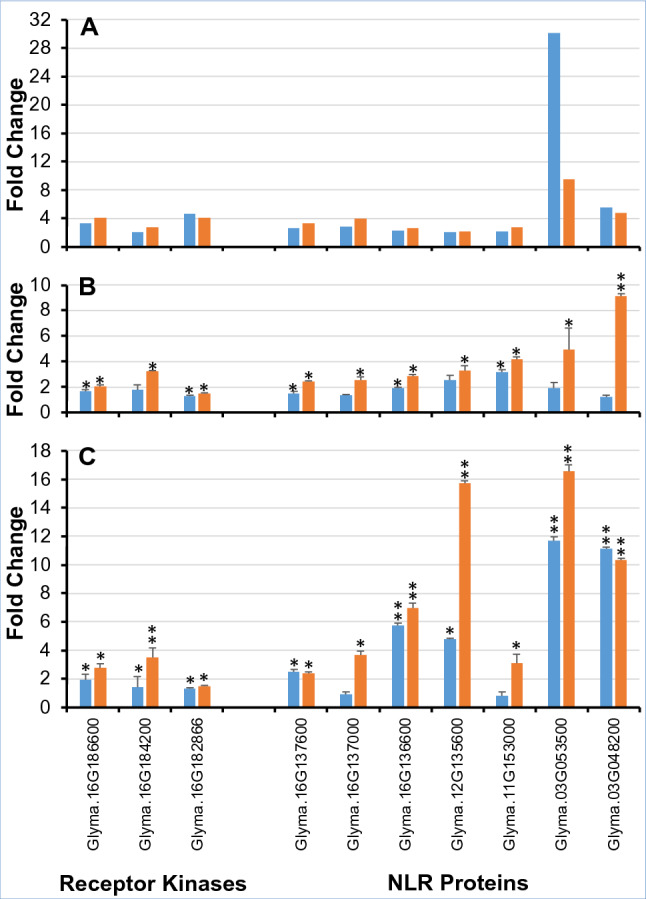
A subset of chitin-responsive genes encoding LRR-receptor kinases and NLR proteins are induced in *GmDR1*-overexpressors following *F. virguiforme* and *H. glycines* infection. (**A**) Significantly enhanced expression levels of LRR-receptor kinases and NLR genes 12 h following chitin treatment (RNA-seq data). Fold changes were calculated by comparing FPKM values of a gene in *GmDR1*-overexpressors, either DR1-136 (blue bars) or DR-107 (orange bars), with that in the nontransgenic control Williams 82 line 12 h following chitin treatment. Transcription fold changes of the genes were at least twofold and *p* ≤ 0.05. (**B**) Significantly enhanced expression levels of receptor kinases and NLR genes 14 days following *F. virguliforme* infection. Fold changes of the genes between *GmDR1*-overexpressors and Williams 82 following *F. virguliforme* infection were determined by RT-qPCR. Data in *B* and *C* are mean fold change ± SE determined for either DR1-136 (blue bars) or DR-107 (orange bars), from three biological replications. The transcript levels of soybean *Elongation factor 1-b* (*ELF1-b*) gene (*Glyma.02g44460*) was used as an internal control in RT-qPCR. *, *p* ≤ 05 and **, *p* ≤ 01. (**C**) Significantly enhanced expression levels of receptor kinases and NLR genes 15 days following *H. glycines* infection. Fold changes of the genes between *GmDR1*-overexpressors, either DR1-136 (blue bars) or DR-107 (orange bars), and Williams 82 following *H. glycines* infection were determined by RT-qPCR. RT-qPCR data of all 28 DEGs are presented on Table S7.

Among the chitin-induced DEGs, the transcript levels of seven defense-related WRKY transcription factor genes were altered (Datasets S2 and S4). In plants, defense mechanisms are modulated by the expression of multiple transcription factors including WRKY proteins, ET-responsive factors (ERFs), and MYB-like proteins^35,37–39^. It was reported that soybean genes encoding WRKY transcription factors were differentially expressed following *Phytophtora sojae* infection^40,41^. Similarly, *F. graminearum* infection induced the expression of more than 10 *BdWRKY* genes in *Brochypodium distachyon*^42^. *WRKY22* is upregulated by chitin in Arabidopsis^43^. Expression of five *GmWRKY* genes enhanced *H. glycines* resistance in soybean roots^34^. Interestingly, among the seven DEGs encoding GmWRKY chitin-induced transcription factors identified in this study, *Glyma.03G042700* and *Glyma.04G223200* were shown to be upregulated following *P. sojae* and *Peronospora manshurica* infection, respectively^41,44^. It was discovered through RT-qPCR analysis that *Glyma.04G223200* is induced in roots of *GmDR1*-overexpressors following *F. virguliforme* and *H. glycines* infection (Fig. 3, Dataset S2). This study also identified four chitin-induced DEGs encoding ERF proteins and three encoding MYB-like proteins. Here again, the data are supported by previous studies that suggested involvement of plant ERF transcription factors in biotic and abiotic defense responses^45,46^. In soybean, *GmERF113* is important for resistance against *P. sojae*^36^; and it was shown that the overexpression of *GmERF3* enhances abiotic stress tolerance and disease resistance in tobacco^47^. It was also shown that the overexpression of *GmERF5* induces immunity of soybean against *P. sojae*^48^. Furthermore, MYB-like proteins are regulators of biotic and abiotic defense responses^37,49^. Thus, characterization of more transcription factors will likely contribute to generate disease resistant crop plants.

The following five highly chitin-induced genes among *GmDR1-*overexpressors belong to protein families putatively involved in plant immunity (Table S4). (1) *Glyma.11G003500* (FC = 25) encoding a pyridoxal-5-phosphate-dependent enzyme family protein with sequence-specific DNA binding domains is putatively involved in biosynthesis of tryptophan, the precursor of biologically essential metabolites that are involved in plant defenses^50^. (2) *Glyma.03G068200* (average FC = 21) encoding a drought-repressed protein is involved in soybean-aphid interactions^51^. (3) *Glyma.03G053500* (average FC = 20) encodes an uncharacterized TNLR protein. (4) *Glyma.03G053602* (FC = 13) encodes an uncharacterized TNLR protein. (5) *Glyma.05G141200* (FC = 13) encoding a jasmonate-zim-domain (JAZ)-like protein is involved in the modulation of plant-pathogen interactions^52^. On the contrary, the five most highly chitin-repressed DEGs encode two proteins of unknown functions, a feruloyl CoA ortho-hydroxylase, a citrate-binding protein*,* and a mitochondrial-processing peptidase (Table S5)*.*

This study also revealed that feeding of phosphate buffer through cut stems of soybean seedlings regulates many genes. Surprisingly, of the 115 DEGs 113 were suppressed and only two were induced in the *GmDR1*-overexpressors as compared to Williams 82 12 h following buffer treatment (Table S6, Dataset S3). This observation suggest that GmDR1 also recognizes damage-associated molecular patterns (DAMP) released from the cut soybean stem and/or buffer-associated cues.

### Identification of the subset of chitin-responsive genes differentially regulated following *F. virguliforme* and *H. glycines* infections

The differential regulation of 23 genes encoding LRR domain containing receptor-like proteins and other immunity-related signaling genes led us to wonder if any of these genes have roles in conferring *GmDR1*-mediated broad-spectrum resistance against pathogens and pests. As a first step towards answering the relevance of these chitin-induced receptors and signaling genes in broad-spectrum resistance, the quantitative reverse transcriptase polymerase chain reaction (RT-qPCR) was conducted to determine if the expression levels of 28 genes encoding nine receptor-kinases, 13 NLR and six factors involved in signaling and metabolic pathways are altered in soybean roots following infection with either *F. virguliforme* or *H. glycines*. The expression data revealed that three receptor protein kinases and seven NLR receptor protein genes were induced in roots of at least the strong *GmDR1*-overexpresser, DR1-107, following *F. virguiforme* and *H. glycines* infection (Fig. 3; Table S7).

### Gene ontology (GO) functional classification of DEGs

To further understand the nature of genes regulated by the overexpressed-*GmDR1* gene, the function of the 192 chitin-responsive, 115 buffer-responsive genes were analyzed using the GO enrichment tool in SoyBase. Upregulated and downregulated DEGs were analyzed separately. As shown in Fig. S3, classification based on cellular component GO term the up-regulated genes are overrepresented in the nucleus (90 genes) followed by either plasma membrane or extracellular region (50 genes) (Fig. S3A). The chitin-responsive upregulated genes were overrepresented in signal transduction (40 genes) and carbohydrate metabolism process (10 genes), when genes were classified based on biological processes (Fig. S3B). The downregulated DEGs were predominately classified to the cell differentiation processes. The downregulated genes were localized to the nucleus (16%), plasma membrane (14%), and cytoplasm (13%) (Fig. S3A). Based on the molecular functions of the genes, most upregulated genes (40 genes) grouped to classes that showed DNA or protein binding activities suggesting that these genes are involved in transcriptional regulation (Fig. S3C).

The 115 buffer-responsive DEGs, regulated by overexpressed-*GmDR1,* were also classified based on GO terms (Fig. S4A–C). The majority (113 genes) of the buffer responsive DEGs were suppressed following buffer treatment in both transgenic lines (Fig. 1D). The proteins encoded by these genes were localized mostly to nuclei, plasma membrane and extracellular region (Fig. S4A) and they showed DNA binding activities based on their molecular functions (Fig. S4C).

### Identification of genes differentially regulated in response to both chitin and buffer treatments

There were only seven genes that were differentially expressed in *GmDR1*-overexpressors as compared to nontrangenic control following both chitin and buffer treatments (Fig. 1A). Three of these seven genes have no known functions (Table S3). The four genes with known functions include *Glyma.02g268200* encoding an aminocyclopropanecarboxylate oxidase involved in ethylene biosynthesis and up-regulated by pyralid feeding^53^, *Glyma.09G131100* encoding heavy metal transport/detoxification superfamily protein responsive to cold and drought stresses^54^; *Glyma.16G021000* encoding a homologue of the homeobox-leucine zipper protein ATHB-12 protein induced by ABA and water stress^55^, and *Glyma.14G171100* encoding the homologue of the transport and Golgi organization 2 protein^56^.

## Discussion

The soybean GmDR1 is an integral plasma membrane protein. It is composed of 73 amino acids with two predicted transmembrane domains and one ectodomain. It is primarily a legume-specific novel protein, homologues of which are also detected in cotton, coca, and jute^22^. *GmDR1* is expressed at a low level and suppressed rapidly during attacks by pathogens and pests^21,22^. Overexpression of *GmDR1* enhances broad-spectrum resistance against pathogens, *F. virguliforme*, *H. glycines*, and pests, spider mites (*T. urticae,* Koch), and soybean aphid (*A. glycines* Matsumura). The chitin, a well-recognized PAMP, is present in all these four organisms, and the transcript levels of the SA- and JA-regulated defense pathway marker genes are induced in *GmDR1-*overexpressors as compared to the nontransgenic Williams 82 control line following treatment with chitin^22^. The chitin could interact directly or indirectly with GmDR1 to induce defense pathways for induction of broad-spectrum resistance. During co-evolution of soybean with its pathogens and pests, pathogens and pests were able to gain mechanisms to suppress the expression of *GmDR1* to cause susceptibility because exchange of the GmDR1 promoter with infection-inducible and strong root- and leaf-specific promoters induced enhanced pathogen and pest resistance^22^.

In this study, chitin was used to determine the responses of the *GmDR1*-overexpressors because it’s a well-recognized PAMP and present in *F. virguliforme*, *H. glycines*, spider mites, and soybean aphid, against which *GmDR1-*overexpressors induced enhanced broad-spectrum resistance. Investigation of the chitin regulated transcriptomes of the two *GmDR1*-overexpressors revealed that chitin enhances expression of defense-related genes in the two overexpressors as compared to the nontransgenic Williams 82 control (Dataset S2). Surprisingly, in addition to the known genes regulating defense responses, 23 genes encoding LRR domain containing proteins including ten receptor kinases and 13 NLR proteins were identified. The transcription levels of genes encoding nine receptor kinases and all 13 NLR proteins were induced and that of one receptor kinase gene, *Glyma.01G062900,* was suppressed following chitin treatment among the *GmDR1*-overexpressors compared to nontransgenic control (Dataset S2).

To determine if any of the chitin-induced nine LRR receptor kinases and 13 NLR proteins may be involved in broad-spectrum immunity, RT-qPCR on RNA samples collected from roots of the *GmDR1*-overexpressors and nontransgenic Williams 82 plants infected with *F. virguliforme* and *H. glycines* was conducted. It was observed that three of the nine DEGs encoding LRR receptor kinases and seven of the 13 DEGs encoding NLR receptor proteins were significantly induced in at least one of the *GmDR1*-overexpressors following *F. virguliforme* or *H. glycines* infection (Fig. 3; Table S7). The soybean Williams 82 genome carries at least 319 NLR genes^11^, of which only a very few have been partially or fully characterized (*Rps11*, *Rps1*-k, *Rps4*, *Rpg1*-b, *Rsv1*)^57–61^. This work has identified several candidate NLR genes that could be involved in pathogen and pest resistance and laid a strong foundation for functional characterization of additional soybean *NLR* genes involved in immunity.

PAMP and microbe associated molecular patterns (MAMPs) are recognized by cell surface/plasma membrane-localized pattern recognition receptors (PRRs), which include receptor-like kinases (RLKs) and receptor-like proteins (RLPs). The PRR, FLAGELLIN SENSING 2 (FLS2) receptor recognizes the PAMP, bacterial flagellin flg22 and activates defense pathways in *Arabidopsis thaliana*^62^. Chitin is a PAMP widely present in many organisms. In rice, CEBiP transmembrane receptor recognizes chitin and induces defense pathways^63^.

PAMPs, MAMPs, host derived damage- or danger-associated molecular patterns (DAMPs) are recognized by cell surface/plasma membrane-localized pattern recognition receptors (PRRs), which include mostly receptor kinases (RKs), receptor-like kinases (RLKs), and receptor-like proteins (RLPs). To date, 69 PRRs (^64–66^; Table S8) have been reported. The 29 of the 69 PRRs were identified from *Arabidopsis* (Table S8). Twelve of the ligands recognized by these receptors are from bacteria, thirteen from fungi, two from viruses, one from aphid, and nine are host derived DAMPs. Among the 17 PRRs identified from *Solanum* species, 14 recognize bacterial ligands, one recognizes a fungal ligand, two recognize DAMPs. Among the 13 PRR from *Oryza* species, seven recognize bacterial and six fungal ligands. From *Lotus*, *Nicotiana*, and *Brassica* species, seven PRRs were detected that recognize bacterial ligands. On the contrary, the two PRRs from *Triticum* species are unidentified orphans that recognize fungal ligands. Only one PRR has been identified from *Glycine,* an orphan protein that recognizes a fungal beta glucan-binding protein (GBP). The 27 of the 69 identified PRRs are RLPs (39%), 21 (30%) are RKs, 16 (23%) are RLKs, and only one is LRR-RP.

From this study, it appears that *GmDR1* regulates several immunity-related receptor kinases and NLR receptors presumably involved in the expression of resistance against diverse pathogens and pests; and therefore, GmDR1 is most likely a key PAMP recognition receptor that induces PTI against both pathogens and pests in soybean. The GmDR1 protein, responsive to chitin and multiple pathogens and pests, is most likely a novel PRR protein and it does not fall into any of the characterized PRR classes reported earlier and represents a member of a new class of PRRs (Table S8).

Induction of a large number LRR domain containing receptor protein genes among the GmDR1-overexpressors is very intriguing. It is hypothesized that only a subset of the chitin-induced 22 receptor protein genes including 13 *NLRs* may regulate downstream defense genes following infection against a specific pathogen or pest. The RT-qPCR data supported this hypothesis. It was observed that three of the nine receptor kinase genes and seven of the 13 *NLR*-type disease resistance receptor protein genes were significantly induced in at least one of the two *GmDR1*-overexpressors as compared to the nontransgenic Williams 82 control following infection with either *F. virguliforme* or *H. glycines* (Fig. 3).

In this study, it is established that the enhanced broad-spectrum disease and pest resistance among the *GmDR1* overexpressors mediated through induced expression of genes involved in plant immunity^67^. GmDR1 most likely recognizes one or more PAMPs from multiple pathogens and pests and activates multiple signaling pathways to provide soybean with immunity against pathogens and pests. It’s therefore most likely a PAMP recognition receptor for inducing PTI against several pathogen and pests; and chitin could be one of the PAMPs recognized by GmDR1. This transcriptomic study also indicates that GmDR1 may recognize other environmental ques including DAMPs as indicated by the responses of the *GmDR1* overexpressors to phosphate buffer.

Induction of nine and repression of one LRR-receptor protein kinases is also intriguing and it is possible that one or more of these receptor kinases may partner with GmDR1 containing only 73 amino acids during recognition of molecular patterns-specific to pathogen, pest, or tissue-damages. This study most importantly revealed that the broad-spectrum immunity induced by the overexpression of *GmDR1*could be mediated by 13 NLR proteins, a subclass of which was found to be induced by two soybean pathogens. NLRs are involved in the expression of ETI. This study indicates that NLRs could also be involved in the expression of PTI. Alternatively, GmDR1-induced NLRs may be involved in effector triggered immunity against the two pathogens. Requirement of PTI in the expression ETI has recently been reported^68^. The possible activation of ETI pathway by a pattern recognition receptor GmDR1 for PTI and/or ETI certainly very intriguing. It suggests an interdependence between PTI and ETI and priming of ETI by PTI for effective use of the cellular defense mechanisms for expression of broad-spectrum basal as well as gene- or race-specific disease resistance against multiple pathogens.

### Supplementary Information

Supplementary Information.

## Data Availability

The RNA-seq data are available at https://www.ncbi.nlm.nih.gov/geo/query/acc.cgi?acc=GSE226254.
