# Supplementary material for: The soybean plasma membrane GmDR1 protein conferring broad-spectrum disease and pest resistance regulates several receptor kinases and NLR proteins
Source: Sci Rep. 2024 May 28;14:12253. doi: 10.1038/s41598-024-62332-4 (PMC11133457; doi:10.1038/s41598-024-62332-4)
Supplement: Supplementary file 1 — Supplementary Information. [file 41598_2024_62332_MOESM1_ESM.docx]

*Tetranychus urticae*, Koch **Supporting Information**

**This file includes:**

Tables S1-S8

Figures S1 to S4

**Other supplementary materials for this manuscript include the following:**

Datasets S1 to S4

**Table S1**. Primers used in reverse transcriptase - quantitative polymerase chain reaction (RT-qPCR).

| **Name** | **Sequence** | **Amplicon size (bp)** |
| --- | --- | --- |
| Glyma.01G062900-F | GCTATGGGGATAACTGCTGTGATC | 178 |
| Glyma.01G062900-R | CAACACCAGAACACCCCTTCCC |  |
| Glyma.03G048200-F | CCTGAATTAGAAACTCTGATTGTTAAAG | 177 |
| Glyma.03G048200-R | GGACTGTAAGGAGTTGGCAGTTTCTTG |  |
| Glyma.03G048500-F | GAGTTAAGGAGGTTGCGTAACCATCCAC | 156 |
| Glyma.03G048500-R | GCAATGGTGGTCTTTCCGATACCACC |  |
| Glyma.03G053500-F | GCCTACGGTTACGACGACTACGATATCC | 214 |
| Glyma.03G053500-R | CCCCTTGGAAGTGTCTCATCATCCTTG |  |
| Glyma.03G053602-F | GGGACCATCTCTCTTCAAGTAGAATC | 198 |
| Glyma.03G053602-R | CAATAAGCGCTTAATCAAGCCTTTTTTTG |  |
| Glyma.03G066800-F | GATTGCAGAGAGATTGGGGAAGACACC | 269 |
| Glyma.03G066800-R | CTGGCCTTAACTAGCCTTTCCTGC |  |
| Glyma.04G057700-F | GGAATACAAGTGTGAGATGAAGGTTACCC | 186 |
| Glyma.04G057700-R | CATAGTAGTAGAACATGACGAGGCAGTGG |  |
| Glyma.04G223200-F | GGTTCATGAAACGACCACACAGCAGTC | 177 |
| Glyma.04G223200-R | CTCAGAATCTTCCCCATCTTTCCCTCC |  |
| Glyma.05G126400-F | CCAACTTCAAAACATGGAGAAGCTACTTTGC | 159 |
| Glyma.05G126400-R | GAGGAAATTGTGAGGGTCTCTAGTG |  |
| Glyma.07G077700-F | CTGTTCCTGACGTCACCTCGTTTAAAGGC | 170 |
| Glyma.07G077700-R | CTCTCCCACGTCGATGTTCTTCCTCGTC |  |
| Glyma.09G075500-F | GTGCCAGATTATGCACTGCCACAAGG | 185 |
| Glyma.09G075500-R | CCTTGTGGCAGTGCATAATCTGGCAC |  |
| Glyma.10G148700-F | GCCAATATCTTGCGTGAGAGAGATGG | 169 |
| Glyma.10G148700-R | GAAATTGGTGGCACAACTTTGGCTCCC |  |
| Glyma.11G003500-F | GCTGTGCTCACTACTCATCAGGAACC | 182 |
| Glyma.11G003500-R | GGTCAAAGAAGCTACAAGTGTCTCAGGTAC |  |
| Glyma.11G153000-F | CCAACCGTTGTTACGATTGGTCAGTTCAG | 165 |
| Glyma.11G153000-R | GGCCCATGGTCACCTTGTATACAGC |  |
| Glyma.12G135600-F | GCA CTC AAA CAT CGC CAG GAA GTG TTC | 170 |
| Glyma.12G135600-R | CCCACTTCTTTCAGGGCTTCTCTCC |  |
| Glyma.13G028100-F | GGAGGGATCAGTCTTAAAAGATAACCGG | 180 |
| Glyma.13G028100-R | CAGAAATGATCATACACTTCACTTATTCCGCC |  |
| Glyma.14G118800-F | GATGGTTGTGGCCTCTCAAGGTACG | 163 |
| Glyma.14G118800-R | CGAGGAATGGAGCAAGCAGTATCCTC |  |
| Glyma.16G136600-F | GACCGTTTCCATGATGGCTGCTTCAG | 150 |
| Glyma.16G136600-R | GCATTCGTCCCACGACAGGTTACATAC |  |
| Glyma.16G137600-F | GCATGGAGGATATTCGATTCAGTGATCC | 178 |
| Glyma.16G137600-R | CAACTTGATTCCTCTTTCATCCACACGTGTGAG |  |
| Glyma.16G137000-F | CCACCGTTGAAGTTGACCTCTCTTGAAAGGC | 157 |
| Glyma.16G137000-R | CTCGGCTTCGATCCTTATGTCTTGGACCC |  |
| Glyma.16G182700-F | CACCTCGACCTCTCTGGTACTGGATTC | 173 |
| Glyma.16G182700-R | GGTTCGACAACAGAATGACCTCCAAGGCC |  |
| Glyma.16G182866-F | GGCAAATCCTTCTTTCCTTTGTGCAATG | 167 |
| Glyma.16G182866-R | GATTCCCAATCTGAGATGGAATTGTTCC |  |
| Glyma.16G184200-F | GGAAGTGATGGGCATGGAGTGAATTGG | 186 |
| Glyma.16G184200-R | CAATTTGGAAAGAAACACACCTGAGCGAAAAG |  |
| Glyma.16G186100-F | GATTTATATGCAGAACCAACAACTACACTTG | 155 |
| Glyma.16G186100-R | GTGTCTCACGCTCACTTGGGATGC |  |
| Glyma.16G186600-F | CCTCCTCCATTTTTTATATTCTTGTCTTTGTCC | 167 |
| Glyma.16G186600-R | GTTGGTATTATTATGATTCCAAGACC |  |
| Glyma.16G193600-F | GGAAAATCTTGAGATTGACTTGATGGTAG | 163 |
| Glyma.16G193600-R | GTGGCAGAGGACTCCATACCAGTGGC |  |
| Glyma.17G011700-F | GGATCGCCGGTAGACTCAACCC | 189 |
| Glyma.17G011700-R | GGGTGTTCCAGTTGTTTCTTTGTTCGC |  |
| Glyma.17G180300-F | GAATTG AAAGGCGTGTGTTCGCCTCC | 160 |
| Glyma.17G180300-R | CCAACGTTGACTTCCCTGAGCCTGG |  |
| Glyma.18g254000-F | CCATATGGTTTCAGTGGGAGTGAGC | 161 |
| Glyma.18g254000-R | GACCAGCTGATTGTGCTTGTGCC |  |

**Table S2**. Chitin-responsive genes induced in the nontransgenic Williams 82 line12 h following chitin treatment.

| **Gene name** | **Fold Change** | **Annotation** |
| --- | --- | --- |
| Glyma.03G201500 | 5.40 | Late embryogenesis abundant protein |
| Glyma.14G102900 | 5.11 | WRKY DNA -binding domain |
| Glyma.02G132500 | 3.80 | AP2 domain |
| Glyma.04G223300 | 3.66 | WRKY DNA -binding domain |
| Glyma.17G222500 | 3.16 | WRKY DNA -binding domain |
| Glyma.17G222300 | 2.88 | WRKY DNA -binding domain |
| Glyma.06G020800 | 2.44 | Unknown protein |
| Glyma.05G150100 | 2.30 | Unknown protein |
| Glyma.02G040700 | 2.30 | Mitochondrial carrier protein |
| Glyma.10G170900 | 2.29 | Tyrosine phosphatase family |
| Glyma.03G201100 | 2.24 | Late embryogenesis abundant protein |
| Glyma.04G178500 | 2.20 | Unknown protein |
| Glyma.02G009500 | 2.18 | Unknown protein |
| Glyma.17G128900 | 2.09 | EF hand |
| Glyma.17G053600 | 1.98 | Unknown protein |
| Glyma.17G076600 | 1.95 | SNARE domain |
| Glyma.08G044400 | 1.93 | Calmodulin binding protein-like |
| Glyma.11G096600 | 1.87 | No apical meristem (NAM) protein |
| Glyma.14G061200 | 1.85 | Hs1pro-1 protein C-terminus; Hs1pro-1 N-terminus |
| Glyma.05G023100 | 1.81 | SNARE domain |
| Glyma.03G201000 | 1.76 | Late embryogenesis abundant protein |
| Glyma.02G311200 | 1.75 | Protein tyrosine kinase |
| Glyma.01G128100 | 1.73 | WRKY DNA -binding domain |
| Glyma.03G138000 | 1.66 | Protein kinase domain; EF hand |
| Glyma.10G045400 | 1.60 | Unknown protein |

Fold changes are statistically significant at *p* < 0.05.

**Table S3.** Seven genes differentially expressed between transgenic lines and nontransgenic Williams 82 line 12 h following either chitin or buffer treatment (Fig. 1A).

| **Gene name** | **Average Fold Change** | | **Annotation** |
| --- | --- | --- | --- |
|  | **Chitin** | **Buffer** |  |
| Glyma.02g268200 | -26.37 | 11.47 | 1-aminocyclopropane-1-carboxylate oxidase |
| Glyma.05g142900 | 145.92 | 18.28 | Putative transmembrane protein |
| Glyma.09G131100 | 81.73 | 302.31 | Heavy metal-associated isoprenylated plant protein 39 |
| Glyma.13G266900 | 134.23 | 7.06 | Hypothetical protein |
| Glyma.13G267100 | -38.42 | 14.63 | Uncharacterized protein LOC100305673 |
| Glyma.14G171100 | 24.77 | 3.99 | Transport and Golgi organization 2 homolog |
| Glyma.16G021000 | -30.78 | 2.27 | Homeobox-leucine zipper protein ATHB-12 |

Fold changes are statistically significant at *p* < 0.05.

**Table S4.** Thirty-five genes most-highly induced in both DR1-136 and DR1-107 12 h following chitin treatment (Dataset S2).

| **Gene name** | **Average Fold Change** | **Annotation** |
| --- | --- | --- |
| Glyma.11G003500 | 25.24 | Pyridoxal-5\'-phosphate-dependent enzyme family protein |
| Glyma.03G068200 | 21.10 | Drought-repressed 4 |
| Glyma.03G053500 | 19.78 | Disease resistance protein (TIR-NBS-LRR class) |
| Glyma.03G053602 | 13.38 | TMV resistance protein N-like isoform X2 |
| Glyma.05G141200 | 12.75 | Jasmonate-zim-domain protein 8 |
| Glyma.03G083600 | 9.68 | Mitochondrial substrate carrier family protein |
| Glyma.12G231300 | 8.82 | Uncharacterized protein |
| Glyma.14G116400 | 8.50 | Structural maintenance of chromosomes flexible hinge |
| Glyma.14G118800 | 8.08 | Receptor serine/threonine kinase, putative |
| Glyma.05G236233 | 7.89 | Plant cadmium resistance 2 |
| Glyma.05G236167 | 7.05 | Plant cadmium resistance 2 |
| Glyma.16G186100 | 6.41 | Disease resistance family protein / LRR family protein |
| Glyma.15G015200 | 6.08 | Mitochondrial substrate carrier family protein |
| Glyma.09G182200 | 5.76 | Protein kinase superfamily protein |
| Glyma.10G191700 | 5.67 | Peroxidase superfamily protein |
| Glyma.09G006700 | 5.47 | F-box plant-like protein, putative |
| Glyma.13G267300 | 5.42 | Uncharacterized protein |
| Glyma.16G182700 | 5.40 | Disease resistance family protein / LRR family protein |
| Glyma.09G054100 | 5.37 | Ankyrin repeat family protein |
| Glyma.03G048200 | 5.19 | Leucin-rich repeat containing; NB-ARC domain-containing disease resistance protein |
| Glyma.06G058400 | 5.00 | Integrase-type DNA-binding superfamily protein |
| Glyma.07G113800 | 4.84 | Ethylene-responsive element binding factor 13 |
| Glyma.04G057700 | 4.78 | Integrase-type DNA-binding superfamily protein |
| Glyma.02G132500 | 4.73 | Redox responsive transcription factor 1 |
| Glyma.03G074167 | 4.56 | Uncharacterized protein |
| Glyma.03G111700 | 4.52 | Ethylene-responsive element binding factor 13 |
| Glyma.10G148700 | 4.51 | Calmodulin-binding protein |
| Glyma.04G223200 | 4.50 | WRKY DNA-binding protein 55 |
| Glyma.15G242050 | 4.47 | Uncharacterized protein |
| Glyma.16G182866 | 4.40 | Disease resistance family protein /LRR receptor-like serine/threonine-protein kinase GSO1 |
| Glyma.13G006700 | 4.35 | Nudix hydrolase homolog 2 |
| Glyma.03G048351 | 4.33 | Abc2 homolog 14 |
| Glyma.17G159000 | 4.30 | Phytosulfokine 5 precursor |
| Glyma.12G146700 | 4.23 | Diacylglycerol kinase 5 |
| Glyma.10G226200 | 4.20 | Pyridoxal phosphate (PLP)-dependent transferase |

**Table S5.** Thirty-five genes most highly repressed (FC > 3) in both DR1-136 and DR1-107 following chitin treatment (Dataset S2).

| **Gene name** | **Average Fold Change** | **Annotation** |
| --- | --- | --- |
| Glyma.07G124400 | 6.73 | Feruloyl coa ortho-hydroxylase 1; 1-aminocyclopropane-1-carboxylate oxidase like 1 |
| Glyma.15G011000 | 6.07 | Uncharacterized protein loc100802656 precursor |
| Glyma.13G362800 | 6.01 | Uncharacterized protein loc100780674 precursor |
| Glyma.06G149120 | 5.90 | 24 kda seed coat protein precursor; citrate-binding protein |
| Glyma.15G020800 | 5.71 | Mitochondrial-processing peptidase subunit alpha |
| Glyma.02G215100 | 5.34 | Protein SICKLE |
| Glyma.18G268900 | 5.33 | Longifolia 1 isoform x1 |
| Glyma.13G309600 | 5.28 | Uncharacterized protein loc100527444 |
| Glyma.09G204800 | 4.76 | Glycine-rich cell wall structural protein 1.8 |
| Glyma.03G058800 | 4.63 | Uncharacterized protein loc100779876 |
| Glyma.10G200800 | 4.59 | Cytochrome p450 monooxygenase cyp76o2; 7-ethoxycoumarin o-deethylase-like precursor |
| Glyma.17G080500 | 4.31 | Cytochrome P450 78A5 |
| Glyma.14G169700 | 4.16 | Sulfate transporter 1.3 isoform x1 |
| Glyma.18G197400 | 3.99 | Transcription factor nai1 |
| Glyma.12G176200 | 3.86 | Hypothetical protein |
| Glyma.07G007102 | 3.83 | Lipoxygenase-10 |
| Glyma.12G109800 | 3.81 | (R,s)-reticuline 7-o-methyltransferase |
| Glyma.08G175400 | 3.78 | Nicotianamine synthase |
| Glyma.01G062900 | 3.64 | Leucine-rich repeat receptor-like protein kinase family |
| Glyma.07G186100 | 3.60 | Putative branched-chain-amino-acid aminotransferase 7 |
| Glyma.10G092800 | 3.47 | Probable 2-oxoglutarate-dependent dioxygenase aop1 |
| Glyma.18G106300 | 3.36 | Laccase-4 isoform x1 |
| Glyma.07G016800 | 3.35 | Gata transcription factor 9 |
| Glyma.15G243300 | 3.31 | Cytochrome P450 |
| Glyma.12G179100 | 3.29 | Histone H2B.3 |
| Glyma.04G035600 | 3.27 | Cytochrome P450 82C4 |
| Glyma.09G238300 | 3.22 | Gibberellin-regulated protein 14 precursor |
| Glyma.18G269800 | 3.18 | Uncharacterized protein loc100500577 precursor |
| Glyma.07G149600 | 3.16 | Vacuolar cation/proton exchanger 3 |
| Glyma.04G058800 | 3.15 | Putative glutamine dumper family protein |
| Glyma.15G131500 | 3.12 | Endoribonuclease dicer homolog 2 isoform x1 |
| Glyma.08G262800 | 3.10 | Alpha-xylosidase 1 |
| Glyma.18G103400 | 3.07 | Spermidine hydroxycinnamoyl transferase |
| Glyma.11G153800 | 3.05 | Annexin-like protein rj4 isoform x1 |
| Glyma.17G074600 | 3.05 | Reticulon-like protein b21 isoform x1 |

**Table** **S6.** Thirty-five genes most-highly repressed following buffer treatment (Dataset S2).

| **Gene name** | **Average Fold Change** | **Annotation** |
| --- | --- | --- |
| Glyma.08G341100 | 88.81 | Kunitz-type trypsin inhibitor precursor |
| Glyma.11G119200 | 44.96 | Transmembrane protein |
| Glyma.04G062600 | 40.36 | Zinc finger protein 1 |
| Glyma.06G177600 | 37.55 | Uncharacterized LOC111255648 |
| Glyma.10G072400 | 37.21 | Uncharacterized protein LOC100780937 |
| Glyma.13G286600 | 35.29 | Thioredoxin protein |
| Glyma.11G046000 | 29.00 | Phytosulfokines |
| Glyma.12G051800 | 25.45 | Fructokinase-2 |
| Glyma.03G215400 | 25.10 | Alpha/beta-Hydrolases superfamily protein; epoxide hydrolase A isoform A |
| Glyma.12G031300 | 23.31 | Nuclear fusion defective 4 |
| Glyma.02G264800 | 21.55 | RING-H2 finger protein ATL66 |
| Glyma.18G228600 | 19.79 | Peptidoglycan-binding lysm domain-containing protein |
| Glyma.15G228400 | 19.67 | Hypothetical protein |
| Glyma.03G163200 | 18.67 | Uncharacterized protein LOC100776152 |
| Glyma.16G051800 | 18.37 | NAC domain containing protein 83 |
| Glyma.15G060400 | 18.25 | Proline/glycine/tyrosine-rich protein |
| Glyma.15G068900 | 17.89 | Glycine-rich cell wall structural protein 2 |
| Glyma.14G221800 | 17.30 | E3 ubiquitin-protein ligase ATL23 |
| Glyma.09G041900 | 16.98 | Uncharacterized protein LOC100305812 |
| Glyma.17G001800 | 16.33 | Protein NRT1/ PTR FAMILY 4.3 [Glycine max] |
| Glyma.09G196700 | 14.62 | RING-H2 finger protein ATL8 |
| Glyma.02G130400 | 13.03 | Chalcone synthase 10 |
| Glyma.12G155200 | 12.65 | Hypothetical protein |
| Glyma.10G186800 | 12.57 | Ethylene-responsive transcription factor 1B |
| Glyma.11G231400 | 12.54 | RING-H2 finger protein ATL66 |
| Glyma.11G254600 | 12.49 | Rapid alkalinization factor (RALF)-like 24 |
| Glyma.09G190300 | 12.47 | Protein TIC 214 |
| Glyma.11G077800 | 12.43 | Thaumatin-like protein 1 pathogenesis-related protein |
| Glyma.07G240900 | 12.22 | Uncharacterized protein LOC113002203 |
| Glyma.02G039100 | 11.74 | Leucine-rich repeat-containing protein 1 |
| Glyma.13G367600 | 11.70 | Sterile alpha motif (SAM) domain-containing protein |
| Glyma.06G067700 | 10.86 | Auxin-responsive protein IAA28 |
| Glyma.08G232500 | 10.79 | Bifunctional inhibitor/lipid-transfer protein/seed storage 2S albumin superfamily protein |
| Glyma.07G104300 | 10.55 | Uncharacterized protein LOC111138524 |
| Glyma.05G247800 | 10.05 | Purple acid phosphatase 3 |

**Table S7.** A subset of genes encoding NB-LRR proteins, receptor kinases, signaling and defense genes are induced in *GmDR1*-overexpressers following chitin treatment, *Fusarium virguiforme (F.v.)* and soybean cyst nematode (SCN) *Heterodera glycines* infection.

| **Gene name** | **RNA-seq**  FC (p-value ≤ 0.05) | | ***Fusarium virguliforme* (Fv) infection**  FC | | | | | **Soybean cyst nematode (SCN) infection**  FC | | | | |
| --- | --- | --- | --- | --- | --- | --- | --- | --- | --- | --- | --- | --- |
|  | 136/W82 Chitin | 107/W82 Chitin | 136/W82 Infection | 107/W82 Infection | 136/W82 Control | 107/W82 Control | W82Infection/ W82Control | 136/W82 Infection | 107/W82 Infection | 136/W82 Control | 107/W82 Control | W82Infection/ W82Control |
| **Receptor kinases** | | | | | | | | | | | | |
| Glyma.18G254000 | 3.25 | 2.85 | 0.85 | 2.67 | 0.73 | 0.63 | 2.77 | 1.98 | 3.95* | 1.92 | 1.74 | 1.58 |
| Glyma.16G193600 | 2.34 | 3.51 | 1.27* | 1.37* | 1.25 | 1.02 | 1.14 | 1.14 | 1.22 | 1.76 | 1.76 | 1.33 |
| Glyma.16G186600 | 3.28 | 4.12 | 1.63* | 2.07* | 1.18 | 0.91 | 0.88 | 1.95* | 2.79* | 2.24 | 1.70 | 1.38 |
| Glyma.16G186100 | 2.95 | 9.87 | 1.73* | 2.25* | 1.33 | 1.01 | 0.87 | 0.44 | 0.59 | 2.46 | 1.53 | 0.69 |
| Glyma.16G184200 | 2.11 | 2.74 | 1.81 | 3.23** | 1.06 | 0.88 | 0.74 | 1.42 | 3.49* | 0.15 | 0.26 | 1.59 |
| Glyma.16G182866 | 4.69 | 4.10 | 1.28* | 1.50* | 1.14 | 0.96 | 1.01 | 1.32* | 1.50* | 1.56 | 1.33 | 1.32 |
| Glyma.16G182700 | 5.48 | 5.33 | 1.00 | 1.06 | 1.06 | 1.06 | 1.07 | 1.00 | 0.98 | 1.20 | 1.11 | 1.24 |
| Glyma.14G118800 | 4.34 | 11.83 | 1.07 | 4.67** | 1.58 | 0.99 | 3.04** | 1.64 | 1.65 | 1.01 | 0.95 | 1.57 |
| Glyma.05G126400 | 3.07 | 3.25 | 1.06 | 1.08 | 1.01 | 1.02 | 1.09 | 1.00 | 1.04 | 1.16 | 1.04 | 1.16 |
| **Nucleotide-binding Leucine-Rich Repeat (NB-LRR) proteins** | | | | | | | | | | | | |
| Glyma.17G180300 | 2.36 | 2.81 | 4.62 | 6.78 | 4.41* | 0.21 | 0.58 | 0.89 | 0.30 | 0.51 | 1.24 | 1.24 |
| Glyma.16G137600 | 2.63 | 3.26 | 1.45* | 2.40* | 1.47 | 1.38 | 1.23 | 2.49* | 2.40* | 1.35 | 1.31 | 1.45 |
| Glyma.16G137000 | 2.85 | 4.01 | 1.37 | 2.56* | 0.69 | 0.63 | 2.76* | 0.90 | 3.64* | 1.59 | 0.91 | 1.06 |
| Glyma.16G136600 | 2.30 | 2.67 | 1.93* | 2.88* | 0.42 | 0.88 | 1.87 | 5.75** | 6.99** | 0.88 | 0.51 | 1.68 |
| Glyma.13G028100 | 2.42 | 3.07 | 1.39 | 2.22* | 1.29 | 1.28 | 3.05* | 0.47 | 0.54 | 0.76 | 0.67 | 0.78 |
| Glyma.12G135600 | 2.00 | 2.13 | 2.53 | 3.31* | 1.28 | 1.90 | 3.35* | 4.77* | 15.70** | 0.50 | 0.46 | 1.16 |
| Glyma.11G153000 | 2.11 | 2.74 | 3.16* | 4.15* | 0.84 | 0.68 | 1.93 | 0.79 | 3.09 | 1.25 | 0.86 | 1.88 |
| Glyma.09G075500 | 2.13 | 2.03 | 0.00 | 0.00 | - | - |  |  |  |  |  |  |
| Glyma.07G077700 | 2.19 | 2.20 | 1.84 | 1.39 | 1.64 | 2.03 | 1.05 | 2.19 | 0.94 | 1.33 | 1.95 | 2.99 |
| Glyma.03G053602 | 18.81 | 7.95 | 0.00 | 0.00 | - | - |  |  |  |  |  |  |
| Glyma.03G053500 | 30.10 | 9.45 | 1.92 | 4.91 | 1.13 | 0.63 | 1.84 | 11.67** | 16.57** | 3.09* | 4.08* | 0.81 |
| Glyma.03G048500 | 4.99 | 3.05 | 0.00 | 0.00 | - | - |  |  |  |  |  |  |
| Glyma.03G048200 | 5.57 | 4.81 | 1.20 | 9.12** | 0.47 | 0.48 | 1.84 | 11.15** | 10.34** | 0.46 | 0.85 | 0.82 |
| **Signaling proteins** | | | | | | | | | | | | |
| Glyma.17G011700 | 2.62 | 2.41 | 1.84 | 1.39 | 1.11 | 0.89 | 10.43** | 4.03* | 24.26** | 0.51 | 0.44 | 0.61 |
| Glyma.11G003500 | 5.25 | 45.23 | 1.60 | 1.53 | 1.89* | 1.68 | 2.67* | 2.37 | 0.74 | 0.74 | 2.30 | 1.15 |
| Glyma.10G148700 | 2.94 | 6.08 | 0.75 | 1.53 | 0.48 | 0.42 | 2.34* | 0.75 | 1.53 | 0.69 | 0.97 | 2.03* |
| Glyma.04G223200 | 2.61 | 6.39 | 1.36 | 26.71** | 0.57 | 0.58 | 4.56* | 1.33 | 3.32* | 2.19 | 1.13 | 1.56 |
| Glyma.04G057700 | 3.07 | 6.48 | 2.84 | 2.64 | 1.34* | 2.41 | 3.39 | 7.04** | 4.61* | 0.58 | 1.09 | 2.64* |
| Glyma.03G066800 | 2.22 | 2.39 | 1.92* | 8.86** | 1.13 | 0.66 | 11.41** | 1.89 | 1.35 | 1.02 | 1.99 | 1.55 |

**RNA-seq:** Fold changes (FC) of selected 28 DEGs in *DR1-136* (136) and *DR1-107* (107) were compared to Williams 82 (W82) 12 h following chitin treatment. FCs were calculated by comparing FPKM values of a gene in either *DR1-107* or *DR1-136* with that in the nontransgenic control W82 line. All fold changes are at least 2-fold and significant at *p* = 0.05.

***F.v.* infection**: FC of the same 28 DEGs between *GmDR1*-overexpressers and W82 in roots following *F.v.* infection and with no infection, and between W82 following *F.v*. infection and with no infection were determined by RT-qPCR.

**SCN infection**: FC of the same 28 DEGs between *GmDR1*-overexpressers and W82 in roots following SCN infection and with no infection, and between W82 following SCN infection and with no infection were also determined by RT-qPCR.

Data of *F.v* and SCN infections are mean determined from three biological replications. The soybean *Elongation factor 1-b* (*ELF1-b*) gene (*Glyma.02g44460*) was used as an internal control in RT-qPCR. *, *p* ≤ 05 and **, *p* ≤ 01.

**Table S8**. PAMP or DAMP (PRR) recognition receptors reported earlier (Zipfel, 2014; Saijo et al., 2018; and DeFalco et al., 2021).

| **Organism** | **Classes of PRR** | | **Pathogens** |
| --- | --- | --- | --- |
|  | ***Type*** | ***Number*** | ***Type (number)*** |
| Arabidopsis | LRR-RK | 8 | Bacteria (2)  Arabidopsis (3)  Unidentified (3) |
|  | LRR-RP | 1 | Bacteria (1) |
|  | LRR-RLK | 4 | Bacteria (1)  Damage associated patterns (3) |
|  | LRR-RLP | 4 | Bacteria (1)  Fungi (2)  Orphan (1) |
|  | LysM-RLK | 1 | Bacteria (1) |
|  | LysM-RLP | 3 | Bacteria (3) |
|  | LysM-RK | 2 | Fungi (2) |
|  | LEC-RLK | 3 | Bacteria (2)  DAMP (1) |
|  | LecRK | 1 | Unidentified (1) |
|  | GPI-AP | 1 | Arabidopsis (1) |
|  | EGF-like-RLK | 1 | DAMP (1) |
| Solanum | LRR-RK | 3 | Bacteria (3) |
|  | LRR-RLK | 2 | Solanum (1)  DAMP (1) |
|  | LRR-RLP | 9 | Bacteria (8)  Fungi (1) |
|  | LysM-RLK | 2 | Bacteria (2) |
|  | LEC-RLK | 1 | Bacteria (1) |
| Oryza | LRR-RK | 3 | Bacteria (3) |
|  | LRR-RLK | 1 | Bactria (1) |
|  | LRR-RLP | 8 | Bacteria (3)  Fungi (5) |
|  | LEC-RK | 1 | Unidentified from fungi (orphan) (1) |
| Lotus | LysM-RK | 2 | Bacteria (2) |
|  | LysM-RLK | 1 | Bacteria (1) |
| Nicotiana | LRR-RK | 1 | Bacteria (1) |
|  | LRR-RLP | 1 | Bacteria (1) |
| Brassica | LRR-RLP | 2 | Bacteria (2) |
| Triticum | RK | 1 | Unidentified from fungi (orphan) (1) |
|  | RLK | 1 | Unidentified from fungi (orphan) (1) |
| Glycine | Beta-glucan-binding protein | 1 | Fungi (orphan) (1) |

**
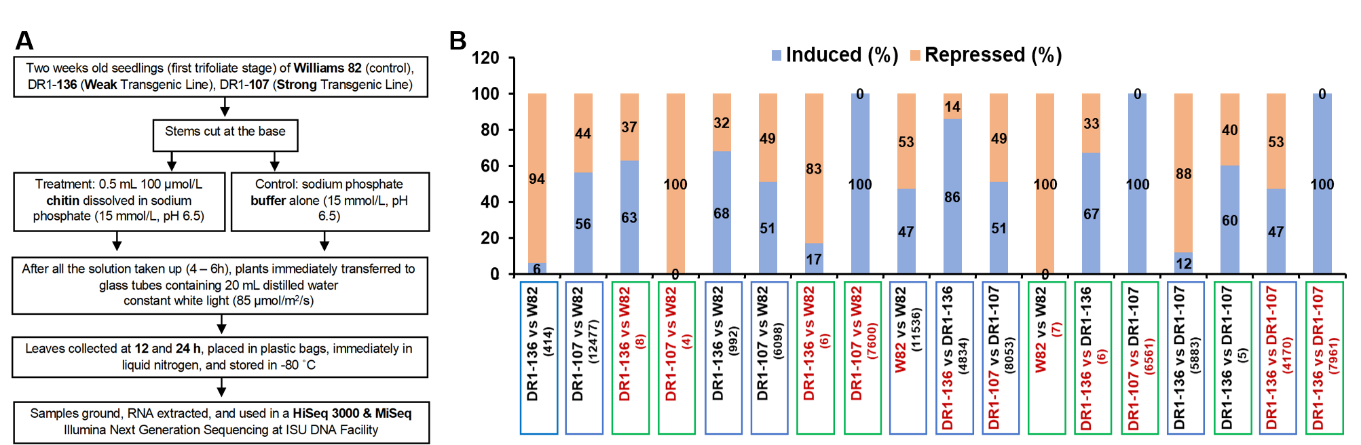
**

**Figure S1.** Transcriptomic study of the soybean leaves of Williams 82 and *GmDR1*-overexpressers DR1-136 (weak) and DR1-107 (strong) following chitin and phosphate buffer treatments. *(A)* Summary of the steps taken to obtain the RNA-seq data. *(B)* Comparative analysis of differentially expressed genes (DEGs) (log2(FPKMy/FPKMx) 0.05 0r higher and *p value* 0.1 between Williams 82 (W82) and *GmDR1*-overexpressers DR1-136 (weak) and DR1-107 (strong). The number of DEGs are shown in parentheses. Data presented on the bar graph are percentages of DEGs (Dataset S1). Letters and numbers in Black Font on the X-axis legend show the data of buffer treatment and those with Red Font are for the chitin treatment. Information in blue boxes on the X-axis legend represents data of 12 h and those in green rectangles for 24 h following treatments.


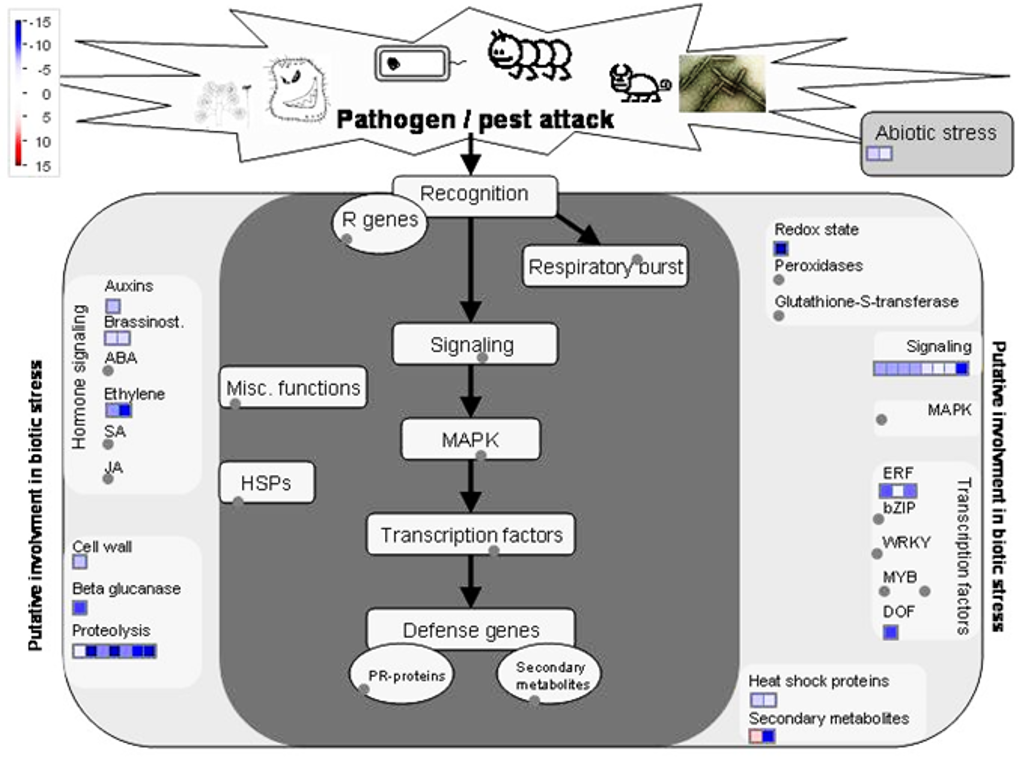


**Figure S2. Genes regulated by overexpressed *GmDR1* following treatment with phosphate buffer.** From the 115 buffer-responsive genes, 28 involved in the abiotic and biotic-stress pathways (Dataset S4C) and seven in metabolic pathways (Dataset S4D) were identified. Blue indicates a decrease, whereas red indicates an increase (log2 fold changes) in the transcription levels of a gene when compared with that of the nontransgenic Williams 82 control.


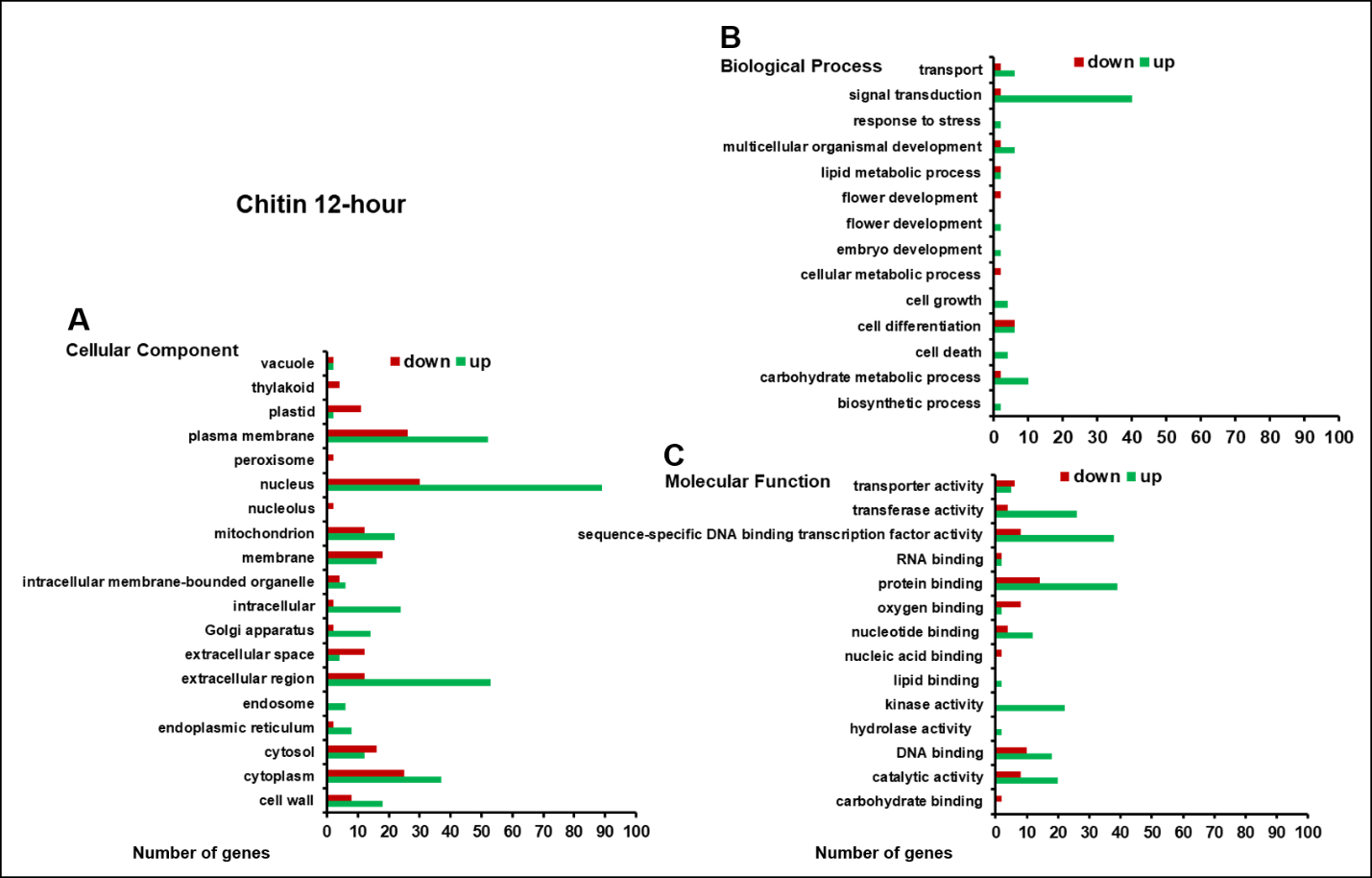


**Figure S3.** Functional classification of GO terms of 192-chitin responsive genes (Dataset S2) differentially expressed in both transgenic lines 136 and 107 as compared to Williams 82 12-hour following chitin treatment (Fold Change ≥ 2; *p* ≤ 0.001). The classifications based on (A) Cellular component, (B) Biological process, and (C) Molecular function of the characterized DEGs are presented.


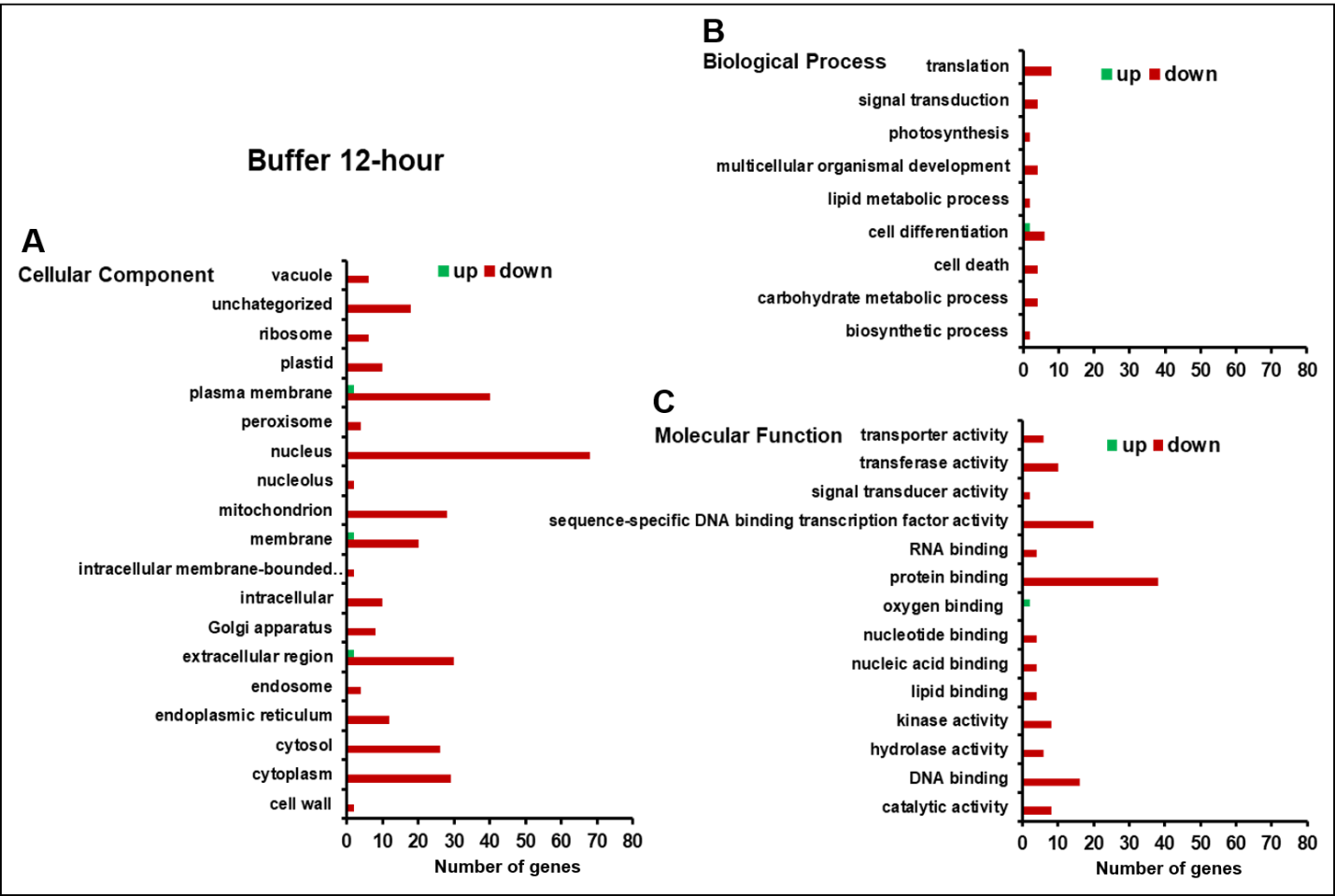


**Figure S4.** Functional classification of GO terms of 115-buffer responsive genes (Dataset S3) similarly differentially expressed in both transgenic lines 136 and 107 vs Williams 82 12-hour following phosphate buffer treatment (Fold Change ≥ 2; *p* ≤ 0.001). The classifications based on (A) Cellular component, (B) Biological process, and (C) Molecular function of the characterized DEGs are presented.

**Legends for Datasets S1-S4**

Dataset S1. Number of genes differentially expressed in transgenic lines compared to controls.

Dataset S2. Genes differentially expressed between Williams 82 and transgenic lines at 12 h following chitin treatment.

Dataset S3. Genes differentially expressed between Williams 82 and transgenic lines at 12 h following buffer treatment.

Dataset S4. Mapman analysis of differentially expressed genes between W82 and transgenic lines.

Dataset S4A. Chitin-induced genes involved into the biotic stress pathway.

Dataset S4B. Chitin-induced genes involved into the metabolism pathway.

Dataset S4C. Buffer-responsive genes involved into the abiotic and biotic stress pathway.

Dataset S4D. Buffer-responsive genes involved into the metabolism pathway.
